# Lycopodium Mitigates Oxidative Stress and Inflammation in the Colonic Mucosa of Acetic Acid-Induced Colitis in Rats

**DOI:** 10.3390/molecules27092774

**Published:** 2022-04-26

**Authors:** Salim M. A. Bastaki, Naheed Amir, Ernest Adeghate, Shreesh Ojha

**Affiliations:** 1 Department of Pharmacology and Therapeutics, College of Medicine and Health Sciences, United Arab Emirates University, Al Ain P.O. Box 15551, United Arab Emirates; naheedmiramir@gmail.com (N.A.); shreeshojha@uaeu.ac.ae (S.O.); 2 Department of Anatomy, College of Medicine and Health Sciences, United Arab Emirates University, Al Ain P.O. Box 15551, United Arab Emirates; eadeghate@uaeu.ac.ae

**Keywords:** lycopodium, IBD, colonic inflammation, gut, oxidative stress, plants

## Abstract

Inflammatory bowel diseases (IBDs) such as ulcerative colitis (UC) and Crohn’s disease (CD) are diseases of the gastrointestinal system involving genetic and environmental factors attributed to oxidative stress and inflammation. Targeting oxidative stress and inflammation by novel dietary compounds of natural origin convincingly appears to be one of the important therapeutic strategies to keep the disease in remission. As there is no permanent cure for IBD except for chronic long-term treatment or surgery, it is therefore imperative to investigate plant-based agents that are receiving attention for their therapeutic benefits to overcome the debilitating clinical conditions of IBD. Lycopodium (LYCO), a plant of tropical and subtropical origin and known by numerous names such as ground pine, club moss, or devil’s claw, has been popularly used for centuries in traditional medicine including Chinese and Indian medicines. In the present study, the effect of LYCO has been investigated in an acetic acid (AA)-induced colitis model in Wistar rats. LYCO was orally administered at the dose of 50 mg/kg/day either 3 days before or 30 min after the induction of IBD and continued for 7 days by intrarectal administration of AA. The changes in body weight and macroscopic and microscopic analysis of the colon of rats of different experimental groups were observed on days 0, 2, 4, and 7. The levels of myeloperoxidase (MPO), reduced glutathione (GSH), and malondialdehyde (MDA) were measured. AA caused a significant reduction in body weight and increased macroscopic and microscopic ulcer scores along with a significant decline in antioxidant enzymes, superoxide dismutase (SOD), and catalase and antioxidant substrate, glutathione (GSH). There was a concomitant increased formation of malondialdehyde (MDA), a marker of lipid peroxidation, and raised myeloperoxidase (MPO) activity, a marker of neutrophil activation. Treatment with LYCO significantly improved IBD-induced reduction in body weight, improved histology, inhibited MDA formation, and restored antioxidants along with reduced MPO activity. AA also caused the release of proinflammatory cytokines such as interleukin-1β (IL-1β) and interleukin-23 (IL-23). Furthermore, AA also increased the levels of calprotectin, a protein released by neutrophils under inflammatory conditions of the gastrointestinal tract. LYCO treatment significantly reduced the release of calprotectin and proinflammatory cytokines. The results demonstrate that LYCO treatment has the potential to improve disease activity by inhibiting oxidative stress, lipid peroxidation, and inflammation along with histological preservation of colonic tissues.

## 1. Introduction

Inflammatory bowel diseases (IBDs), such as ulcerative colitis (UC) and Crohn’s disease (CD), are characterized by low-grade chronic inflammation and immune dysregulation of the intestine with alternating relapses and remission and require long-term treatments [1]. Its etiology is unknown, although it is considered a group of complex heterogeneous gastrointestinal diseases involving genetic variations, intestinal microbiota, immunological factors, and environmental factors, which interact with an inherent genetic predisposition [2,3]. The interaction between these factors leads to the disruption in the balance between pro- and anti-inflammatory components and are among the key causes of intestinal inflammation in IBD [4]. Inflammation is often accompanied by oxidative stress caused by overproduction of reactive oxygen and nitrogen species, which play central roles in the pathophysiology of IBD [5]. The products of oxidative stress along with intensifying inflammation contribute to the destruction of cell structure by lipid peroxidation, eventually causing cell apoptosis and necrosis. Thus, oxidative stress and inflammation were shown to be key players in the pathogenesis of IBD and represent important therapeutic targets for IBD [5,6,7,8,9].

The available therapies for IBD are far from satisfactory for the majority of patients who require continuous medication to keep the disease under control [10]. In addition, the currently used agents including amino salicylates, corticosteroids, immunomodulators, and biological agents often have various side effects with variable responses and can diminish over time as well as limit their therapeutic benefits [11]. To date, no cure aside from radical surgery for refractory ulcerative colitis and no preventive measures are available for these conditions. The use of plants in disease management goes back thousands of years and is based on traditional consumption in diets for medicinal benefits [12,13]. In recent years, numerous plant extracts and phytochemicals have been shown to ameliorate intestinal inflammation through different molecular pathways, including anti-inflammatory and immunoregulatory mechanisms, antioxidative properties, and modulation of intracellular signaling transduction pathways [8,9,12,13,14,15]. The use of plant-based drugs as complementary and alternative medicines has gained popularity among patients with IBD (30–50%), not only due to their dissatisfaction with the existing IBD therapies but also due to their pharmacological properties attributing to the attenuation of oxidative stress and inflammation as well as correcting nutritional deficits and preventing complications [13,16,17].

Among the numerous plants used in the treatment of intestinal inflammatory diseases, a plant, Lycopodium (LYCO) has garnered attention due to its potent multiple properties including immunomodulation. LYCO (synonymous Huperzia), family *Lycopodiaceae*, a plant of tropical and subtropical forests, is also known by other names such as club moss, or devil’s claw or ground pine, and constitutes an important component in traditional medicine for renal diseases, rheumatic arthritis, cystitis, and gastritis [18]. LYCO has been demonstrated to possess anticancer [18,19], antioxidant [20,21], and anti-inflammatory [22,23] properties. LYCO has been well characterized to contain alkaloids and triterpenoids as major constituents, and these compounds have received attention for their potential pharmacological properties [24,25]. To test its therapeutic potential in gastrointestinal diseases, LYCO nano-formulations have been observed experimentally to exhibit potent anticancer activity against colon cancer by inhibiting cell growth and enhancing cell death [19,25]. A recent preliminary study demonstrated that the constituents of *Lycopodium clavatum* elicit potent anti-inflammatory activity, suggesting its usefulness in IBD [24,25]. In another study, LYCO showed potent immunomodulatory actions on digestive pathogenic progression in Chagas disease wherein the infection involved cytokine-mediated inflammatory reactions [22]. There is no report available to date on the effect of LYCO in ulcerative colitis. Thus, considering the antioxidant and anti-inflammatory role of LYCO, it is worthwhile to investigate the efficacy of LYCO against colitis induced in rats using acetic acid (AA), a classical model widely employed to imitate the pathophysiologic process of IBD and investigate preventive and therapeutic agents [8,26].

In this study, the protective effects of LYCO on the macroscopic and microscopic pathologies were investigated in the rat colon. Additionally, the activities of antioxidant enzymes and the levels of proinflammatory cytokines and calprotectin were measured in rat colon tissues.

## 2. Experimental Animals

Before conducting the animal experiments, written permission (animal ethics approval no. ERA_2019_6000) was obtained from the Institutional Animal Ethical Committee of the United Arab Emirates University, and all experimental procedures were carried out in accordance with ethical guidelines on animal studies. Male adult albino Wistar rats, weighing 225–240 g, were housed in polycarbonate cages at standard animal house conditions viz. photoperiod of 12 h light/dark cycle was used for the experiments. The room temperature ranged between 21 and 24 °C and relative humidity between 40 and 60%. The animals were maintained at the animal research facility, located at the College of Medicine and Health Sciences, United Arab Emirates University, Al Ain, United Arab Emirates. The animals were fed commercially available standard chow diet and fed water ad libitum. They were randomly divided into different animal groups and acclimatized for a week before commencing the experiments. Prior to the induction of colitis, the animals were fasted for 24 h. To induce colitis, 1 mL of 4% AA was administered intrarectal at 8 cm proximal to the anus for 30 s under the influence of light anesthesia with ether. To prevent the leakage of the solution, the rats were kept in Trendelenburg position during rectal instillation for a minute. One ml of phosphate-buffered saline was also administered to flush the colon in similar conditions.

### 2.1. Chemicals and Kits

Acetic acid (AA) was obtained from BDH Prolabo (Johannesburg, South Africa). The test drug, lycopodium, was procured from Sigma-Aldrich (St. Louis, MO, USA). The protein assay kit, BCA, and protease inhibitors were procured from Thermo Fisher Inc. (Waltham, MA, USA). Malondialdehyde assay kit and MPO kit were obtained from Hycult Biotech (Uden, The Netherlands) and Northwest Life Science (Vancouver, WA, USA). The IL-1β kit DuoSet and GSH assay kit were obtained from R&D Systems (Minneapolis, MN, USA) and Sigma-Aldrich (Sigma Chemical Co., St. Louis, MO, USA), respectively. The kits for calprotectin and IL-23 were obtained from MyBioSource Inc. (San Diego, CA, USA). Sodium deoxycholate and all other chemicals that were not specified were obtained from Sigma-Aldrich (St. Louis, MO, USA).

### 2.2. Experimental Design

**Figure 1:**
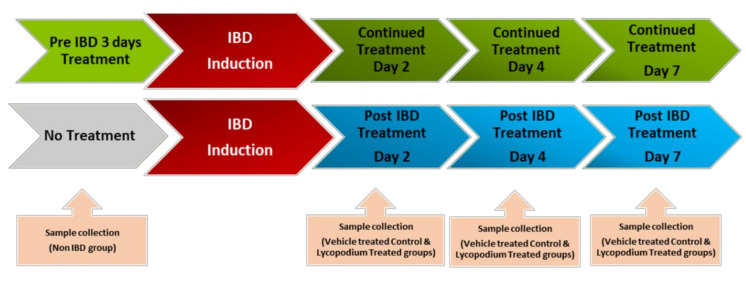
Schematic representation of study design.

The schematic representation of the study design is presented in Figure 1. The doses of LYCO were chosen on the basis of a pilot study-assessed dose response from our previously published laboratory studies [20]. The dose of 50 mg/kg prepared in olive oil was administered orally once a day using an intragastric gavage. The rats were lightly anaesthetized with ether after overnight fasting, and 1 mL of 4% AA was used to induce IBD.

LYCO was given under two different regimes: (1) a therapeutic regimen wherein LYCO effects were tested for 1 week after the induction of IBD and (2) a preventive regimen wherein LYCO was given 3 days before induction of IBD and thereafter for 7 days. Briefly, for each dosing regimen, rats were grouped into pre-IBD LYCO-treated (*n* = 18) and post-IBD LYCO-treated groups (*n* = 18). LYCO was given orally every day 30 min after the induction of IBD assigned as post-treated groups. The same protocol was used in control animals (olive oil alone). In assessing the protective effect of LYCO on IBD, the animals that were administered LYCO 3 days before induction of IBD were assigned as pretreated groups. 

The weight of the animals was taken on days 0 (baseline), 2, 4, and 7 after IBD, and the colon was collected from each group, control, post-treatment, and pre-treatment, and processed for macroscopic, biochemical, and histological assessment and stored accordingly in liquid nitrogen or buffered formalin (4%).

### 2.3. Assessment of Macroscopic Ulcer Score

The colon damage was assessed by macroscopic and microscopic observations. The samples for histological analysis were collected at days 0 (baseline), 2, 4, and 7 days of IBD with or without LYCO treatment. The rats were euthanized by cervical dislocation after 7 days of IBD. The colon was excised 2 cm above the anal margin following longitudinal opening and washed with saline. Macroscopic injury was examined based on scores following the method described by Jo et al. [25], wherein the area of inflammation and the presence or absence of ulcers were observed [27]. 

The assessing standards for macroscopic injury followed semiquantitative score wherein the observations were graded numerically as follows: no ulcer, no inflammation (0); no ulcer, local hyperemia (1); ulceration without hyperemia (2); ulceration and inflammation one site only (3); ulceration and inflammation on two or more sites (4); ulceration extending more than 2 cm (5).

### 2.4. Histopathology Study

The colon histology was examined after staining with hematoxylin and eosin on days 0, 2, 4, and 7 after IBD induction in rats treated with or without LYCO. Colon samples were retrieved from rats of each experimental group and processed according to the standardized method. In brief, the rats used for the experiment were sacrificed following cervical dislocation, and surgery was performed to remove the colon in proximity to the anal opening.

The colon samples were trimmed of adherent connective tissue and fixed in phosphate-buffered saline-buffered formalin (4%) for a week. They were later washed in tap water for 2 h, dehydrated in graded alcohol, and paraffin embedded following a previously described method [28]. Using a microtome, the tissue was sectioned (5 µm) and examined after hematoxylin-eosin staining. The scoring system adapted from a previously described method was used to grade the degree of mucosal damage in LYCO-treated IBD rats compared to untreated controls [29].

### 2.5. Preparation of Colon Tissue Homogenate

The colon samples of 8 cm length were opened longitudinally and rinsed well with chilled phosphate-buffered saline. After weighing the tissue, homogenization was performed using 28 mm ceramic beads by a bead raptor homogenizer (Omni International, Kennesaw, GA, USA). The homogenizing buffer was ten volumes of chilled high potassium chloride lysis buffer (10 mM Tris-hydrochloric acid, pH 8.0, 140 M sodium chloride, 1M EDTA, 0.5% sodium deoxycholate, 300 mM potassium chloride, and 0.5% Triton X-100) with protease inhibitor cocktail. The homogenates were incubated for 30 min on ice and thereafter centrifuged at 4 °C for 30 min with 15,000 rpm. The obtained supernatant was kept at −40 °C until ELISA was performed. The concentration of protein in each sample was measured using a commercially available kit following a bicinchoninic acid (BCA) protein assay kit (Pierce, Thermo Scientific, Rockford, IL 61101, USA). The content of test markers was normalized to protein content and reported as per mg protein.

### 2.6. Determination of Myeloperoxidase (MPO)

Myeloperoxidase (MPO) activity was determined following the manufacturer’s instructions of sandwich ELISA kits. Briefly, at room temperature, a 96-well microtiter plate, coated with primary antibody, the sample (100 µL), and standards (100 µL) were added for 1 h. After washing, the biotinylated trace antibody was added at a volume of 100 µL in each well for one hour at room temperature. Following washing to bind/interact with biotinylated trace antibodies, the streptavidin-peroxidase conjugate was added for 1 h at room temperature. The plate was again washed to add TMB-ELISA substrate for 30 min at room temperature. Oxalic acid was added to stop the enzyme reaction. After adding a stop solution, the absorbance was measured at 450 nm using a microplate reader (Tecan Group Ltd., Männedorf, Switzerland). The data were presented as ng/mg of colonic protein.

### 2.7. Determination of Calprotectin

Calprotectin in colon samples was determined using a commercially available sandwich ELISA kit (MyBioSource, San Diego, CA, USA). Briefly, to a coated 96 microtiter plate, samples (50 µL) and standards (50 µL) and HRP (100 µL) were added for an hour at 37 °C. Following washing and addition of TMB-ELISA substrate, the absorbance was measured at 450 nm using a microplate reader (Tecan Group Ltd., Männedorf, Switzerland), after adding a stop solution. Calprotectin levels were presented as ng/mg of protein.

### 2.8. Measurements of Reduced Glutathione (GSH), Superoxide dismutase (SOD), and Catalase

Reduced glutathione (GSH) content in colon homogenates was determined following the manufacturer’s instruction of the commercially available kits. To a coated 96-well microtiter plate, the sample and standards were added for 30 min at 37 °C. The absorbance was measured at 450 nm using a microplate reader (Tecan Group Ltd., Männedorf, Switzerland). The data were presented as µM/mg of tissue. SOD assay was performed according to the manufacturer’s protocol. This colorimetric assay utilized the tetrazolium salt for detection of superoxide radicals generated by xanthine oxidase and hypoxanthine. One unit of SOD enzyme was required to inhibit 50% dismutation of the superoxide radical. The inhibition of color development was measured at 450 nm by using Emax Plus microplate reader (Molecular Devices, San Jose, CA 94089, USA). Results are expressed in units per mg of protein. CAT levels were measured by Catalase Assay Kit which utilized the peroxidatic function of CAT for determination of enzyme activity. The method was based on the reaction of the enzyme with methanol in the presence of an optimal concentration of hydrogen peroxide. The formaldehyde produced was measured spectrophotometrically at 540 nm, with 4-amino-3-hydrazino-5-mercapto-1,2,4-trizazole as the chromogen by using EmaxPlus microplate reader (Molecular Devices, San Jose, CA 94089, USA). CAT activity in tissue homogenates is expressed as nmol/min/mg protein.

### 2.9. Measurement of Malondialdehyde (MDA)

Malondialdehyde (MDA) in the colon samples was measured using a commercially available assay kit based on the principle that MDA reaction with thiobarbituric acid (TBA) forms an adduct: MDA-TBA with absorbance on 532 nm. Briefly, the sample of the deproteinated tissue was mixed with a solution containing ethanol, butylated hydroxytoluene, and phosphoric acid and the resultant mixture was subjected to heat for 60 min at 60 °C. Furthermore, the mixture was kept at room temperature for 15 min. Then, the mixture was centrifuged at 10,000× *g* for 3 min and the pink color supernatant was collected and the optical density was measured at 532 nm. The concentration of MDA is presented as µM.

### 2.10. Determination of Inflammatory Cytokines: IL-1β and IL-23

The determination of IL-1β in colon homogenate was carried out by a commercial DuoSet ELISA kit (R&D Systems Inc., Minneapolis, MN, USA). Briefly, the test samples and standards were added to a primary antibody coated 96-well microtiter plate for 30 min at 37 °C. Subsequently, TMB-ELISA substrate was further added, and the resultant mixture’s optical density was read at 450 nm using a microplate reader (Tecan Group Ltd., Männedorf, Switzerland). IL-1β levels are presented as pg per milligram of protein. Similarly, the level of IL-23 in the colon was determined by a commercially available sandwich ELISA kit (MyBioSource, San Diego, CA, USA). Briefly, to the coated 96-well microtiter plates, the sample and standards were added and incubated for 2 h at 37 °C. The detection reagents, provided in the manufacturer kit labelled as reagent A was added after removal of the samples and incubated for 1 h at 37 °C. Next, the detection reagent B provided in the manufacturer kit was added after washing and incubated for 1 h at 37 °C. Following washing, the TMB-ELISA substrate and stop solution was added and the optical density of the resultant mixture was measured at 450 nm, using a microplate reader (Tecan Group Ltd., Männedorf, Switzerland). The levels of IL-23 are presented as pg per milligram of protein.

### 2.11. Statistical Analysis

The data are presented as mean with standard error of mean (SEM). The data were statistically analyzed using SPSS 23.0 software, and the analysis was performed using an independent t-test to determine the significance of the mean between the groups. The criterion of statistical significance was set on *p* < 0.05.

## 3. Results

### 3.1. Effect of Lycopodium on Body Weight (BW)

**Figure 2:**
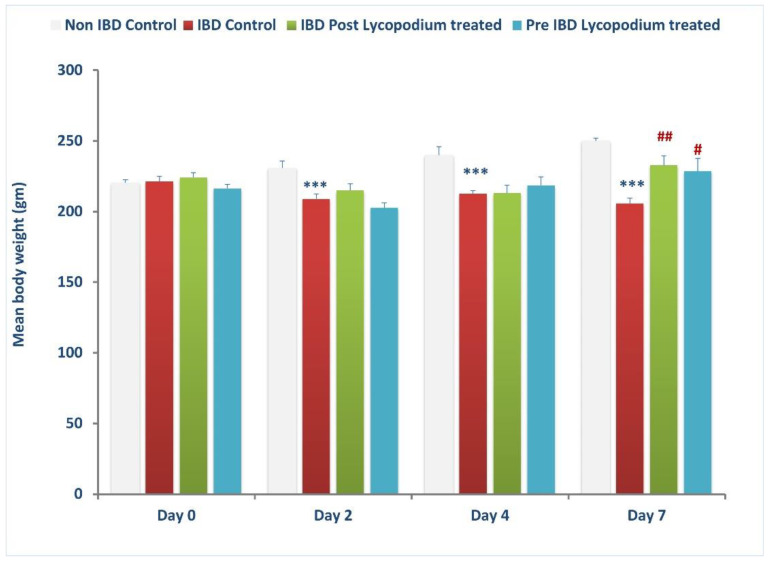
Effect of lycopodium on mean body weight. *** *p* < 0.001 vs. non IBD control group; # *p <* 0.05, ## *p* < 0.01 vs. relative IBD control group.

Figure 2 shows the effect of LYCO on changes in BW of rats. The mean BW in control rats (before IBD) was 220.38 ± 2.0 g (*n* = 6–8, Figure 2). IBD significantly reduced the mean body weight of rats (*p* < 0.001, *n* = 6–9) to 209 ± 3.33 g, 212.5 ± 4.75 and 205.6 ± 3.37 g (*n* = 6–8) on days 2, 4, and 7, respectively. LYCO, given before IBD did not significantly affect BW when compared to IBD control on days 2 and 4 but significantly (*p* < 0.05, *n* = 6–9) increased mean BW from 205.63 ± 3.37 g to 228.63 ± 9.07 g on day 7 of IBD (IBD control). LYCO, given after IBD for 7 days, did not affect the mean BW of rats on days 2 and 4, but produced a significant (*p* < 0.01) increase in the mean BW of rats from 205.63 ± 3.37 to 232.75 ± 6.07 g (*n* = 6–9) on day 7, after the onset of IBD when compared to treated IBD rats (Figure 2). 

### 3.2. Effect of Lycopodium on Macroscopic Ulcer Score (MaUS)

**Figure 3:**
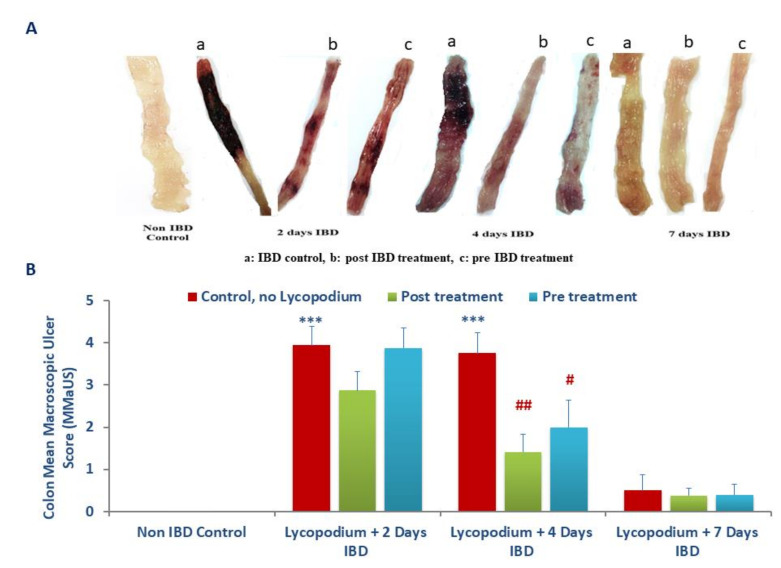
(**A**) Effect of lycopodium on macroscopic image (a: IBD control, b: post IBD treatment, c: pre IBD treatment) and (**B**) ulcer score in rat model of IBD. *** *p* < 0.001 vs. non IBD control group; # *p* < 0.05, ## *p* < 0.01 vs. relative IBD control group.

Figure 3A, 3B show the effect of LYCO on mean MaUS in rats. The mucosa of the control (untreated, no IBD, Figure 3A) was neither hyperemic nor ulcerative, while IBD caused massive hyperemia and ulcer on days 2 and 4 but decreased on day 7 of IBD (3a, 3b, 3c). In the colon, the lesions appeared as hyperemia and ulcers were graded as scores that show a significant (*p* < 0.001) rise in mean MaUS from 0 (control, no IBD, *n* = 6) to 3.94 ± 0.45, 3.75 ± 0.48, and 0.50 ± 0.38 after 2, 4, and 7 days of IBD, respectively. LYCO administered 3 days before or after the induction of IBD produced a subtle reduction in mean MaUS from 3.94 ± 0.45 to 3.88 ± 0.48 (pre-IBD) and to 2.88 ± 0.44 (post-IBD) on day 2 of IBD in comparison with untreated groups (IBD, no LYCO, *n* = 6). However, LYCO administered orally, 3 days before or after IBD, to rats produced a significant (*p* < 0.05, 0.01) decrease in mean MaUS after 4 days of IBD when compared to control (IBD, no LYCO). The mean MaUS decreased from 3.75 ± 0.48 to 1.42 ± 0.42 (post-treatment) and to 2.0 ± 0.63 (pre-treatment) after 4 days of IBD. LYCO did not cause remarkable changes to the mean MaUS on day 7 of IBD.

### 3.3. Effect of Lycopodium on Microscopic Ulcers and Scores (MiUS)

**Figure 4:**
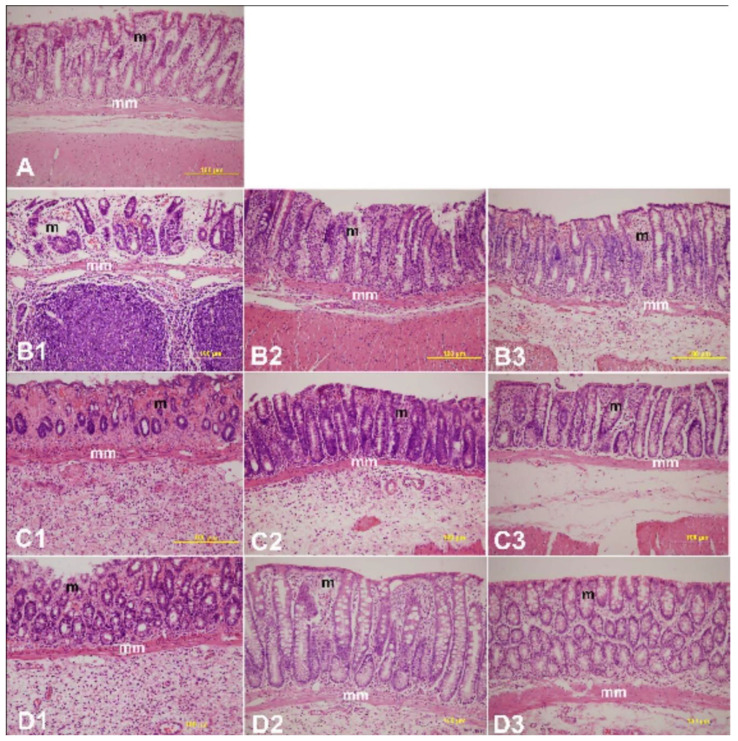

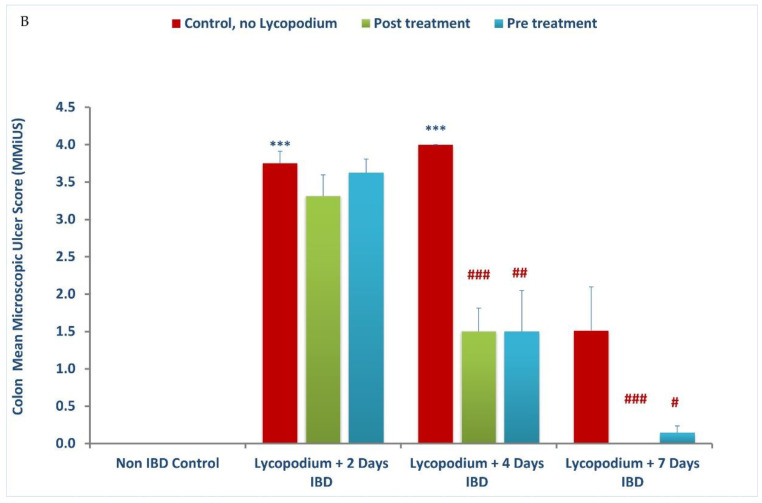
Effect of lycopodium on microscopic images in acetic acid-induced IBD in rats. (**A**): Naïve (control); (**B1**–**B3**): 1: 2-day IBD control–no lycopodium, 2: 2-day IBD, post-treated with lycopodium, 3: 2-day IBD (pre-treated). (**C1**–**C3**): 1: 4-day IBD control–no lycopodium, 2: 4-day IBD, post-treated with lycopodium, 3: 4-day IBD (pre-treated). (**D1**–**D3**): 1: 7-day IBD control–no lycopodium, 2: 7-day IBD, post-treated with lycopodium, 3: 7-day IBD (pre-treated). (**B**) Effect of lycopodium on microscopic ulcer score in a rat model of acetic acid-induced IBD. m= mucosa; mm = muscularis mucosae. *** *p* < 0.001 vs. non IBD control group; # *p* < 0.05, ## *p* < 0.01 ### *p* < 0.001 vs. relative IBD control group.

The effects of LYCO on microscopic histoarchitecture and mean MiUS in the colonic mucosa of control, untreated, and treated rats are presented in Figure 4A,B, respectively. The colon histology of rats administered AA intrarectally displayed severe epithelial loss, submucosal edema, and infiltration of inflammatory cells along with vasculitis. There was a significant (*p* < 0.001) rise in mean MiUS from 0 (untreated) to 3.75 ± 0.16 (*n* = 6), 4.0 ± 0.0 (*n* = 6), and 1.51 ± 0.58 on days 2, 4, and 7 of IBD induction, respectively (Figure 4B). LYCO given 3 days before IBD caused an insignificant reduction in mean MiUS at day 2 [Figure 4A(B1–B3)] but a significant (*p* < 0.05, 0.01) (*n* = 6) reduction in mean MiUS from 4.0 ± 0.0 (IBD, no LYCO) to 1.50 ± 0.55 and from 1.51 ± 0.58 to 0.14 ± 0.09 on days 4 and 7, respectively [Figure 4A(C3,D3)]. Post-treatment of IBD in rats with LYCO significantly (*p* < 0.05, 0.001) reduced mean MiUS from 4.0 ± 0.00 to 1.5 ± 0.31 and from 1.51 ± 0.58 to 0.00 ± 0.00 at days 4 and 7 of IBD [Figure 4A(C2,D2)]. LYCO given after the induction of IBD decreased lymphatic infiltration in the colon (Figure 4A). As shown in Figure 4A, histopathological observations of cross sections of colon of the control group showed normal epithelium with absence of inflammation and necrosis. The administration of AA induced diffused colitis characterized by severe inflammation, goblet cell hyperplasia, edema, and necrosis resembling features of IBD. Pre-treatment with LYCO resulted in partial healing of ulceration and restoration of epithelial and goblet cells with reduction in inflammation and necrosis. Interestingly, histopathological assessment of the colon in LYCO-treated rats revealed healing of the ulcer and mucosal maintenance followed by a remarkable reduction in inflammation and necrosis along with lesser-eroded surface.

### 3.4. Effect of Lycopodium on Myeloperoxidase (MPO) Levels in Colon Tissues

**Figure 5:**
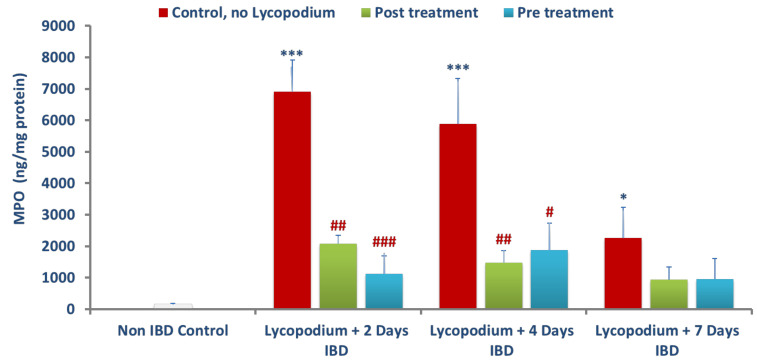
Effect of lycopodium on MPO in acetic acid-induced IBD in rats. Results are mean ± SEM; * *p* < 0.05, *** *p* < 0.001 vs. non IBD control group; # *p* < 0.05, ## *p* < 0.01, ### *p* < 0.001 vs. relative IBD control group.

The effect of LYCO on mean MPO levels, an indicator of neutrophil migration to injured tissue, and the extent of inflammation was measured in colonic tissues, which is presented in Figure 5. The activity of MPO was markedly (*p* < 0.001) increased in the colitis group compared to the control group. Mean MPO in control rats (no LYCO, no IBD) was 143.66 ± 42.6 ng/mg of protein. AA-induced IBD caused a significant (*p* < 0.05, 0.001) increase in MPO to 6907.45 ± 1007.17, 5890.60 ± 1438.66, and 2269.33 ± 961.89 ng/mg of colonic protein in the untreated group (IBD, no LYCO) after 2, 4, and 7 days of IBD. However, LYCO administered post-IBD significantly (*p* < 0.01) (*n* = 6) reduced mean colonic MPO from 6907.45 ± 1007.17 (no LYCO) to 2071.17 ± 271.16 ng/mg of protein and from 5890.60 ± 1438.66 (no LYCO) to 1471.43 ± 390.88 ng/mg of protein at 2 and 4 days of IBD, respectively. LYCO did not affect mean colonic MPO levels after 7 days of IBD. LYCO administered 3 days before IBD produced significant (*p* < 0.05, 0.001, *n* = 6) reduction in mean colonic MPO levels from 6907.45 ± 1007.17 in the untreated group (IBD, no LYCO) to 1121.19 ± 577.30 ng/mg of protein and from 5890.60 ± 1438.66 to 1877.65 ± 850.41 ng/mg of protein, at 2 and 4 days of IBD, respectively. LYCO did not cause any remarkable changes on mean MPO in the colon at day 7 of IBD.

### 3.5. Effect of Lycopodium on Calprotectin Levels in the Colon Tissues

**Figure 6:**
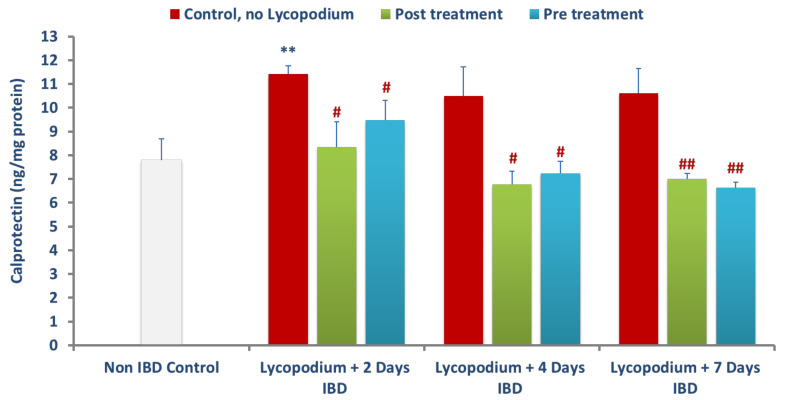
Effect of lycopodium on calprotectin levels in acetic acid-induced IBD in rats. Results are mean ± SEM. ** *p* < 0.01 vs. non IBD control group; # *p* < 0.05, ## *p* < 0.01, vs. relative IBD control group.

The effect of LYCO on calprotectin, a marker of bowel inflammation in colon tissues, is presented in Figure 6. AA caused a significant (*p* < 0.01) increase in the levels of calprotectin from 7.78 ± 0.90 ng/mg of protein (control, no IBD) to 11.40 ± 0.35 ng/mg of protein after 2 days of IBD. There was no significant change in calprotectin levels after 4 and 7 days of IBD. LYCO administered 30 min after IBD significantly (*p* < 0.01, 0.05) decreased mean colon calprotectin levels from 11.40 ± 0.35 to 8.33 ± 1.07 ng/mg of protein, from 10.49 ± 1.21 to 6.76 ± 0.55 ng/mg, and from 10.61 ± 1.01 to 7.00 ± 0.22 ng/mg of protein after days 2, 4, and 7 of IBD. LYCO administered 3 days before IBD produced a significant (*p* < 0.05, 0.001) reduction in calprotectin levels from 11.40 ± 0.35 to 9.46 ± 0.83, from 10.49 ± 1.21 to 7.23 ± 0.50 5, and from 10.61 ± 1.01 ng/mg to 6.63 ± 0.23 ng/mg of protein after 2, 4, and 7 days of IBD, respectively.

### 3.6. Effect of Lycopodium on Interleukin-1 (IL-1β) Levels in Colon Tissues

**Figure 7:**
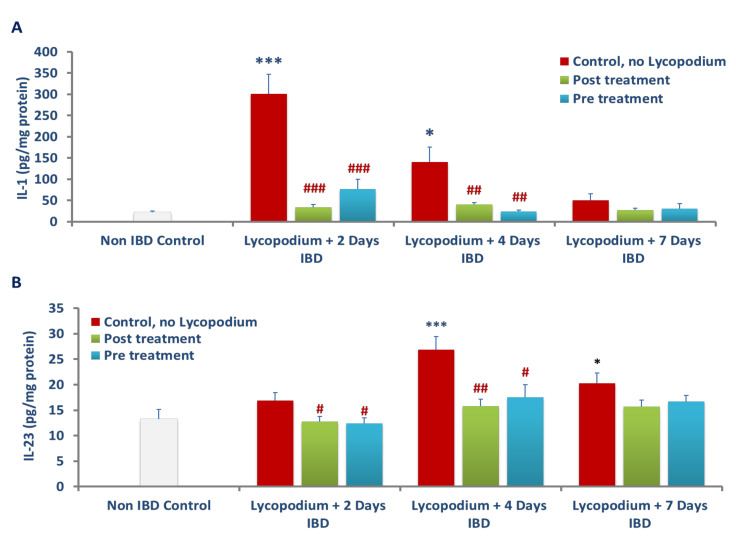
Effect of lycopodium on proinflammatory cytokines; IL-1β (**A**), and IL-23 (**B**) in acetic acid-induced IBD in rats. Results are mean ± SEM. * *p* < 0.05, *** *p* < 0.001 vs. non IBD control group; # *p* < 0.05, ## *p* < 0.01, ### *p* < 0.001 vs. relative IBD control group.

The effects of LYCO on IL-1β, a marker of inflammation in colon tissues are presented in Figure 7A. The IL-1β level in the colon before IBD in the control group (no IBD, no LYCO) was 21.55 ± 3.74 pg/mg of protein. IBD caused significant (*p* < 0.05, 0.001) (*n* = 6) increase in IL-1β levels to 301.30 ± 46.11 (IBD, no LYCO), and 140.78 ± 34.86 pg/gm of protein, on days 2 and 4 of IBD, respectively. On day 7 of IBD, no significant change in IL-1β level was observed. However, LYCO given after IBD produced a significant (*p* < 0.01, 0.001, *n* = 6) reduction in IL-1β levels from 301.30 ± 46.11 (no LYCO) to 34.20 ± 6.18 and from 140.78 ± 34.86 (no LYCO) to 40.38 ± 4.47 ng/mg of protein on days 2 and 4 days of IBD, respectively. When LYCO was given 3 days before IBD, it produced a significant (*p* < 0.01, 0.001, *n* = 6) reduction in the IL-1β levels from 301.30 ± 46.11 in the untreated group (IBD, no LYCO) to 76.90 ± 22.43 ng/mg of protein and from 140.78 ± 34.86 to 24.04 ± 3.34 ng/mg of protein, on days 2 and 4 of IBD, respectively. Pre- and post-LYCO treatment did not significantly change IL-1β levels on day 7 of IBD.

### 3.7. Effect of Lycopodium on Interleukin-23 Levels in Colon Tissues

The effect of LYCO on interleukin-23 (IL-23), another mediator of inflammation in colon tissues, is presented in Figure 7B. In the control group before IBD, IL-23 level (no IBD, no LYCO) was 13.19 ± 1.9 pg/mg of protein. IBD induced a subtle rise in mean IL-23 levels in colon tissues on day 2 with a significant rise from 13.19 ± 1.9 to 26.89 ± 2.5 and 20.25 ± 2.0 mg/mg of protein on days 4 and 7 of IBD, respectively (control, no LYCO, *n* = 6). LYCO given after IBD produced a significant (*p* < 0.01, 0.05, *n* = 6) effect on IL-23 levels in colon tissue on days 2 and 4 of IBD. Mean colonic IL-23 tissue levels decreased from 16.92 ± 1.5 to 12.77 ± 0.9 and from 26.89 ± 2.5 to 15.8 ± 1.3 pg/mg of protein on days 2 and 4 of IBD, respectively. In addition, LYCO given 3 days before IBD produced a significant (*p* < 0.05, *n* = 6) decrease in IL-23 levels from 16.92 ± 1.5 (no LYCO) to 12.39 ± 1.0 from 26.89 ± 2.5 to 17.49 ± 2.5 pg/mg of proteins (*n* = 6) on days 2, and 4 of IBD, respectively. Pre- and post-LYCO treatment has no effect on colonic levels of IL-23 levels on day 7 of IBD.

### 3.8. Effect of Lycopodium on Glutathione (GSH) Levels in Colon Tissues

**Figure 8:**
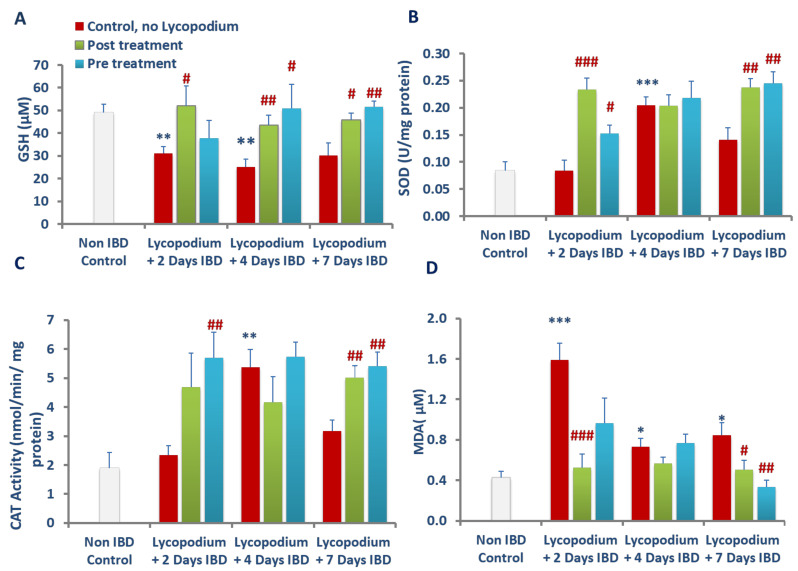
Effect of lycopodium on oxidative stress markers; GSH (**A**), SOD (**B**), Catalase (**C**) and MDA (**D**) in acetic acid-induced IBD in rats. Results are mean ± SEM; * *p* < 0.05, ** *p* < 0.01 *** *p* < 0.001 vs. non IBD control group; # *p* < 0.05, ## *p* < 0.01, ### *p* < 0.001 vs. relative IBD control group.

The effect of LYCO on mean GSH levels in colonic tissues of rats is shown in Figure 8A. Mean colonic GSH level in the control group (no IBD, no LYCO) was 49 ± 3.7 µM (*n* = 6). Mean colonic GSH levels significantly (*p* < 0.01, *n* = 6) decreased to 31.09 ± 2.9 and 25.2 ± 3.5 µM on days 2 and 4 of IBD, respectively. Mean colonic GSH levels non-significantly decreased to 30.24 ± 5.4 µM, on day 7 of IBD. When LYCO was given after IBD, it produced a significant (*p* < 0.05, 0.01) increase in the levels of GSH from 31.1 ± 2.9 to 52.09 ± 8.6, from 25.21 ± 3.5 to 43.55 ± 4.3, and from 30.24 ± 5.4 to 45.78 ± 3.0 µM on days 2, 4, and 7, respectively. LYCO significantly (*p* < 0.05, 0.01, *n* = 6) increased mean colonic GSH levels, when given orally for 3 days before IBD induction. Mean colonic GSH raised was from 25.21 ± 3.5 to 50.89 ± 10.6 and from 30.24 ± 5.4 to 51.52 ± 2.5 µM on days 4 and 7, respectively. LYCO had no effect on mean colonic GSH levels on day 2 of IBD.

### 3.9. Effect of Lycopodium on Superoxide Dismutase (SOD) Activity in Colon Tissues

The effect of LYCO on SOD, an antioxidant enzyme that protects against oxidative stress is presented in Figure 8B. SOD activity before induction of IBD in the control group (no IBD, no LYCO) was 0.08 ± 0.01 U/mg of protein. IBD did not cause significant change in SOD activity on days 2 and 7 of IBD but significantly (*p* < 0.001, *n* = 6) increased mean colonic tissue activity from 0.08 ± 0.01 to 0.20 ± 0.01 U/mg of protein on day 4 of IBD. LYCO given after IBD non significantly increased mean colonic SOD activity on day 4 of IBD but significantly (*p* < 0.01, <0.001, *n* = 6) increased mean colonic SOD activity from 0.08 ± 0.01 to 0.23 ± 0.02 U/mg of protein on day 4 of IBD, and from 0.14 ± 0.2 to 0.24 ± 0.016 on day 7 of IBD. LYCO pre-treatment produced significant (*p* < 0.05, 0.01, *n* = 6) rise in SOD activity from 0.08 ± 0.01 to 0.15 ± 0.01, and from 0.14 ± 0.02 to 0.24 ± 0.02 U/mg on days 2 and 7 of IBD, respectively. Pre- and post-treatment with LYCO had no significant effect on the mean SOD activity in the colon tissue on day 4 of IBD.

### 3.10. Effect of Lycopodium on Catalase Activity in Colon Tissues

LYCO effect on catalase activity, an antioxidative enzyme that removes superoxide anions and thus protects against oxidative stress is presented in Figure 8C. Mean colonic catalase activity before IBD in the control group (no IBD, no LYCO) was 1.89 ± 0.55 nmol/min/mg protein. IBD had no significant effect on mean colonic catalase activity on the days 2 and 7 but significantly (*p* < 0.01, *n* = 6) increased on day 4 of IBD from 1.89 ± 0.55 to 5.38 ± 0.61 nmol/min/mg protein. Post-treatment with LYCO had a subtle effect on mean catalase colonic tissue activity on days 2 and 4, but it had a significantly increased effect on day 7 of IBD. Mean colonic catalase activity increased from 3.17 ± 0.36 to 5.01 ± 0.40 nmol/min/mg protein (IBD, LYCO, *n* = 6). However, when LYCO was administered 3 days before IBD, it remarkably (*p* < 0.01, *n* = 6) increased the mean catalase colonic tissue activity on days 2 and 7 of IBD, from 2.34 ± 0.32 (no LYCO) to 5.72 ± 0.9 and from 3.17 ± 0.36 to 5.41 ± 0.48 nmol/min/mg tissue, respectively. LYCO treatment did not significantly change mean colonic catalase activity on day 4 of IBD.

### 3.11. Effect of Lycopodium on Malondialdehyde (MDA) Levels in Colon Tissues

The effects of LYCO on MDA levels in the colon tissues are presented Figure 8D. IBD caused a significant (*p* < 0.05, 0.001, *n* = 6) rise in the levels of MDA from 0.43 ± 0.06 to 1.58 ± 0.16 and from 0.43 ± 0.06 to 0.73 ± 0.08 and from 0.43 ± 0.06 to 0.84 ± 0.12 µM on days 2, 4 and 7, respectively. LYCO given 30 min after IBD caused a significant (*p* < 0.05, 0.001, *n* = 6) reduction in the levels of MDA from 1.58 ± 0.16 to 0.52 ± 0.16 and from 0.85 ± 0.12 to 0.50 ± 0.08 after 2 and 7 days of IBD. LYCO did not significantly change MDA levels in the colon on day 4 of IBD. LYCO given 3 days before IBD produced a significant (*p* < 0.01, *n* = 6) decrease in MDA levels from 0.84 ± 0.12 to 0.33 ± 0.06 µM on day, but did not significantly change mean MDA levels in colonic tissues on days 2 and 4.

## 4. Discussion

The present study results show that LYCO treatment improved body weight, colonic length, attenuated oxidative stress, and inflammation. Furthermore, the preservation of colonic mucosa clearly indicates the protective and therapeutic effects of LYCO in AA-induced colitis in rats.

Medicinal plants exhibit a variety of pharmacological properties including antioxidant and anti-inflammatory, which shows beneficial effects in IBD. Although many treatment strategies showed beneficial effects for the treatment of IBD, long-term treatments often result in inadequate efficacy and adverse effects. An optimal drug is expected to reduce the disease progression with minimal adverse effects and improve prognosis of the disease. Therefore, in recent years, many research efforts have focused on identifying the health benefits of plants and plant-based medicines in gastrointestinal diseases including IBD [9,30]. This is the first report to demonstrate the effect of LYCO on experimental colitis by suppressing the inflammatory process in the AA-induced rat model of IBD. The AA-induced rat model of IBD is a convenient, economical, reproducible, and commonly used experimental model for evaluating the efficacy of potential drug candidates specifically targeting inflammation and oxidative stress [31]. Enhanced vasopermeability and sustained neutrophil infiltration were seen with this model; in addition, there was an upregulation in inflammatory mediators akin to the pathogenesis of human IBD [26]. The results showed that LYCO modulated mucosal immunity by bringing about a change in the integrity of the epithelial layer and colon injury following neutrophils and macrophage infiltration. In the pathogenesis, leukocytes and granulocytes migrate to the inflamed mucosal layers and the resultant induction and release of proinflammatory cytokines give rise to a hyperinflammatory state that affects the integrity of the colon mucosa and epithelial tissues [32].

In the present study, administration of AA resulted in an increase in proinflammatory cytokines in colon tissues demonstrating the involvement of inflammation in IBD pathogenesis that was further confirmed by histopathology observations displaying neutrophil infiltration, swelling, and necrosis of the epithelial cells [33]. The body weight and colon length of the rats are considered a consistent and sensitive marker of the onset and progression of inflammatory responses [34]. In the current study, maintenance of colon length along with body weight and reduced diarrhea following treatment with LYCO is suggestive of its protective effect in colitis, in line with other studies wherein similar effects were observed with natural antioxidants [8,35,36,37].

Administration of AA resulted in severe colonic inflammation, which was characterized by colon thickening, goblet cell hyperplasia, crypt destruction, ulceration, necrosis following inflammatory cell infiltration and depletion of colonic mucus [26,38]. AA generates protons in the intracellular space leading to high acidification that results in epithelial injury. Treatment with LYCO attenuated the macroscopic and microscopic damages in the rat’s colon as observed in histological studies. The plausible reason for the reduced damage is the preservation of epithelial goblet cells, which is responsible for mucin production. Mucins are the predominant glycoproteins that form a barrier, which protect the bowel against antigens present in the lumen. The vascular mechanisms play an important role in promoting healing and protecting the mucosa in the gut [39,40]. Reduction in gastric blood flow triggers severe gastric ulceration, after the gastric mucosa is exposed to damaging factors [39]. The injury caused to the intestinal mucosa in experimental IBD is convincingly associated with both increased production of free radicals and lesser availability of endogenous antioxidants [6,7].

Under normal conditions, the colonic mucosa has a weaker antioxidant defense relative to other organ systems [41]. In colitis, generation of ROS overpowers the endogenous antioxidant network which keeps a check on free radicals, and ROS becomes imbalanced and leads to oxidative stress and DNA damage [7,8,42]. The role of enzymatic and nonenzymatic antioxidants in protecting tissues from pro-oxidants is well known [9]. The deranged balance in the colonic milieu impairs epithelial cell integrity impedes mucosal recovery and increases intestinal mucosal permeability [34,37]. These changes resulted in impaired function of the mucosal barriers and host defense following weakened endogenous antioxidant defense systems [6,8,42]. The endogenous antioxidant network constitutes nonenzymatic antioxidant substrates, GSH, and enzymatic antioxidants such as SOD, CAT, and GPX to regulate the generation of free radicals and ROS [34,37]. These enzymes protect cells from free radicals by enhancing resistance to oxidative stress, reducing free radicals, and augmenting antioxidants. SOD is the key enzyme that converts oxygen radicals into oxygen and hydrogen peroxide. Furthermore, the enzymes, catalase, and glutathione peroxidase convert hydrogen peroxide into water [34,37]. GSH, an endogenous tripeptide antioxidant, is utilized as a substrate for the enzymatic conversion of hydrogen peroxide into water and oxygen by glutathione peroxidase and plays a critical role in detoxification, increasing amino acid transport, DNA synthesis, and repair [38]. In the present study, LYCO treatment improved SOD and catalase activities and normalized the levels of GSH in colon tissues that demonstrated that LYCO treatment inhibited the excessive generation of free radicals and restored GSH due to its antioxidant properties.

AA-injected animals displayed increased colonic levels of MDA, an indicator of lipid peroxidation following reduced antioxidant defense, and increased formation of lipid peroxides due to lipid peroxidation of polyunsaturated fatty acids in membranes [43]. MDA formation is aggravated by the generation of cytotoxic free radicals destabilizing membrane lipids, proteins, and DNA and plays a role in IBD [37,43]. Furthermore, enhanced lipid peroxidation has been shown in AA-associated tissue injury, which is in line with the observations of the current study. In an earlier study, LYCO was showed to scavenge oxidizing radicals in brain, resulting in an organoprotective effect mediating antioxidant action [20]. Therefore, it has been suggested that the antioxidant and antilipid peroxidation effects of LYCO afford protection from free radicals by restoring the endogenous antioxidants that result in reduced intestinal inflammation and damage in ulcerative colitis. The present study findings are in agreement with previous studies showing a protective effect of plant-derived antioxidants in attenuating inflammation and oxidative injury in experimental models of colitis [35,36,37,43].

The accrual of ROS in ulcerative colonic tissues leads to the onset of inflammation by induction and release of proinflammatory cytokines, IL-1β and IL-23 [8,44,45]. The elevation in the levels of IL-1β and IL-23 in colon tissues indicates the severity and extent of colitis [44]. IL-1β is produced by monocytes and tissue macrophages and plays a critical role in the onset and progression of acute and chronic inflammation at both levels, local and systemic. The synthesis of IL-1β from its immature state is converted into an active state by caspase-1-mediated proteolytic reactions. The epithelial cells and macrophages in the colon release inflammatory cytokines, including IL-1β which contribute to the development of colitis by disrupting the intestinal epithelial barrier, inducing apoptosis of epithelial cells, promoting ROS production, activating neutrophils, and instituting inflammation [44,45]. IL-1β also stimulates the synthesis and release of inflammatory chemotactic proteases, cytokines, and chemokines by activating canonical pathways resulting in the activation of NF-kB, a redox-sensitive transcription factor usually in an inactive state with Ik-B its inhibitor. The stimulation of NF-kB depends on ROS and associated signaling cascades and participates in dereliction of its inhibitor Ik-B following phosphorylation and ubiquitination [44,45]. The NF-kB then activates translocating into the nucleus where its p65 subunit plays a key role in regulating genes controlling the immune-inflammatory cascade. Studies have shown that LYCO suppresses the activation of NF-kB prompted by various inflammatory mediators and exerts genoprotective effects against oxidative and methylation damage in HT-29 colorectal adenocarcinoma cells [25]. In the present study, inhibition of proinflammatory cytokines such as IL-1β and IL-23 in colonic tissues following LYCO treatment demonstrates the anti-inflammatory effect, usually the first step in the treatment of UC.

AA-induced damage to the colon involves neutrophil and macrophage infiltration, and the extent of tissue infiltration is proportionally reflected by the activity of an enzyme, MPO, a measure of neutrophil infiltration [46]. This enzyme is mainly present in azurophilic granules of neutrophils with lesser concentration in monocytes and macrophages and serves as a sensitive and quantitative indicator for acute intestinal inflammation [38,41]. MPO is released from neutrophils during inflammation and facilitates the formation of hypochlorous acid and other free radicals, which exert antimicrobial properties. The free radicals formed cause injury to the host cells by targeting proteins, DNA, and lipids [47]. Neutrophils exhibit a decisive role in the etiopathogenesis of colitis by increasing ROS and superoxide anions that further exacerbate the formation of hydroxyl radicals and peroxide leading to mucosal defense impairment and necrosis. LYCO treatment exhibits a decrease in polymorphonuclear infiltration demonstrated by a significant reduction in MPO activity. In addition to MPO, there was a significant rise in the levels of calprotectin, a protein found in the cytosol of inflammatory cells, following activation of leukocytes that display intestinal inflammation and progression of colitis [48]. Reductions in the levels of calprotectin after treatment with LYCO demonstrate the anti-inflammatory property of LYCO against intestinal inflammation.

The histopathological studies support the macroscopic and biochemical observation on the protective effects of LYCO in colitis. Rectal administration of AA was found to cause tissue ulceration, inflammatory cell infiltration, necrosis, and goblet cell hyperplasia along with reduction in colon length. These observations, which characterize IBD, appear consistent with our previous studies in AA-induced colitis in rats [37]. Pre and post treatment with LYCO significantly improved the colon histoarchitecture by preserving the microscopic and macroscopic structures of the colon and preventing depletion of the colonic wall mucus. Leukocyte infiltration, edema, and tissue injury, which are histological signs of inflammation, appear alleviated following pre-treatment with LYCO. The observation of the present study wherein LYCO protects against colitis is in support of earlier results in its ability to inhibit inflammation and oxidative stress in brain of rats [20]. 

Furthermore, inhibition of NF-kB activation makes it a potentially effective suppressor of cell growth, proliferation, and inflammation [18]. LYCO species are known to contain characteristic quinolizine or pyridine and alfa-pyridone-type alkaloidal and serratene triterpenoid constituents which contribute to the potent antioxidant and anti-inflammatory properties [25,49]. Alkaloids are a class of secondary metabolites in plants containing amino acid-derived nitrogen-containing organic compounds with low molecular weight. Recently, a review highlighted that alkaloid are beneficial in intestinal inflammatory disorders and may provide promising molecules for prophylaxis and treatment of IBD and offer hope for further pharmaceutical development [50]. Similarly, mainly serratene triterpenoids found in LYCO have been shown to exhibit a variety of potent pharmacological properties including antioxidant and anti-inflammatory properties [49]. The serratene triterpenoid compound isolated from LYCO has been recently shown to inhibit inflammatory mediators including enzymes and proinflammatory cytokines in HT-29 cells [25]. It can be reasonably speculated that the triterpenoids and alkaloids attribute to the potential benefits of LYCO in IBD. 

In alternative medicine, the whole plant formulation is considered an effective therapeutic strategy because of their constituents acting synergistically and nutritional properties in chronic low-grade inflammatory diseases including IBD [12]. This concept of using extract particularly reveals a new class of agents, termed as adaptogens, that promote resistance to aversive stimuli perturbing internal homeostasis. The adaptogenic agents have the potential to favorably modulate stress-induced immune and organ impairment by sparing antioxidants and immunomodulation with lesser adverse effects. The plant extract has been found useful for motor abnormalities in children [51]. The use in children is suggestive of the relative safety of LYCO. The results of the present study demonstrate the therapeutic potential of LYCO in IBD and demonstrated the anti-inflammatory and anticolitis activities of LYCO in the experimental model of acute colitis. LYCO administered via the oral route appears effective in numerous disease models and bioavailable with negligible adverse effects that further indicate its potential for nutritional development and its ease of using orally. The results of the present study demonstrate the anti-inflammatory and anticolitis activities of LYCO in the experimental model of acute colitis. 

## Data Availability

The data used to support the findings of this study is already incorporated in the result section.
